# Systematic approach to diagnosis and management of infected prosthetic grafts in the proximal aorta

**DOI:** 10.1111/jocs.15122

**Published:** 2020-11-10

**Authors:** Amer Harky, Ahmed Othman, Carlos Nistal De Paz, Matthew Shaw, Omar Nawaytou, Deborah Harrington, Manoj Kuduvalli, Mark Field

**Affiliations:** ^1^ Department of Cardiac Surgery Liverpool Heart and Chest Hospital Liverpool UK; ^2^ Department of Integrative Biology, Faculty of Health and Life Sciences University of Liverpool Liverpool UK; ^3^ Liverpool Center for Cardiovascular Science University of Liverpool Liverpool UK

**Keywords:** aortic root, Bentall, infection, management

## Abstract

**Objectives:**

Management of infected prosthetic aortic grafts in the ascending and or root is complex and multifaceted. We report our diagnostic pathway, management and outcomes, identifying successful strategies.

**Methods:**

This was a retrospective, single center, observational study. Consecutive patients who underwent management of infected aortic grafts in the ascending and/or root at our institution between October 1998 and December 2019 were included. The main outcome measures were: discharge from hospital alive with at least 1 year survival, operative mortality and success of primary treatment strategy.

**Results:**

Twenty‐six patients presented with infection of proximal aortic grafts and were managed through a number of strategies with an overall hospital‐survival of 81% and 1 year survival of 69%. Twenty of them ultimately underwent redo surgery with 25% operative mortality (within 24 h of surgery). Five patients underwent washout and irrigation of which two were successfully treated and cured with adjunctive antibiotics and two went on to have staged explant and definitive surgery. Interval between surgery and infection was 42.5 ± 35.8 months. All patients had at least one major criterion and three minor criterions with no diagnostic uncertainty. The commonest primary strategy was 3a (definitive surgery), (13/26, 50%).

**Conclusions:**

Adopting a systematic and flexible patient specific approach to the diagnosis and management of patients with proximal aortic graft infections results in reasonable overall 1 year survival. In the majority of patients surgery is ultimately required in an attempt to achieve a curative treatment; however this comes with high operative mortality risk.

## 1 INTRODUCTION

Patients with infected aortic root present a challenge to aortic surgeons as they are not easily eradicated and this gets more complicated when there is an infected prosthetic graft, such as those with previous Bentall root replacement with aortic valve and coronary buttons reimplantation. They can be of a diagnostic challenge and present a complex scenario where careful planning and intervention should be approached through multi‐disciplinary team approach.^1^ Initial treatment with antibiotics can help at early stages and some of these patients may not require surgical intervention; however the ultimate and definitive treatment remains through high‐risk surgical re‐exploration.^2^, ^3^ Several hospitals have published small series of such high‐risk patients including management and outcomes; however, even in major Aortic centers, mortality outcomes are poor and current reported rates are varying between 14% and 55% depending on strategy, timing and approach.^2^, ^3^, ^4^, ^5^ There remains a diagnostic challenge as well as unclear management strategies with little evidence base. We describe a contemporary and pragmatic approach to diagnosis and the management approach developed at Liverpool Heart and Chest Hospital.

## 2 METHODS

We performed a retrospective observational review of our entire practice between October 1998 and December 2019 during which records exist. This study was registered in our institution as a service review, therefore ethical approval and informed consent were not deemed necessary. All patients presenting with infected prosthetic proximal aortic grafts were included. We excluded patients with potential early prosthetic graft infection during the index admission and those sent home on antibiotics following primary surgery. A separate prospective database was collected of patients treated medically. Data for all interventions performed in our center is systematically reported to the National Outcomes for Cardiovascular Outcomes Research and our electronic records have been designed to collect relevant data on all patients undergoing aortic surgical procedures. Data was extracted from this database and included demographics, comorbidity, anatomical and pathological features of aneurysms, morbidity and mortality. The primary outcome measure of the study was in‐hospital mortality. Secondary outcome measures were success of 1 year survival and success of primary treatment strategy.

### 2.1 Diagnostic approach

In diagnosing proximal aortic graft infections we have used two approaches dependent on whether the patient has an isolated proximal graft or proximal graft with prosthetic aortic valve, either aortic valve replacement (AVR) and ascending graft or AVR as part of an aortic root replacement with coronary buttons. Our diagnostic approach has evolved over the duration of the study into a contemporary approach using a combination of Dukes criteria^6^ for diagnosing valve endocarditis and an in‐house modification of the management of aortic graft infection collaboration (MAGIC) guidelines for diagnosing vascular graft infections.^7^

#### 2.1.1 Isolated graft infections

**Figure 1:**
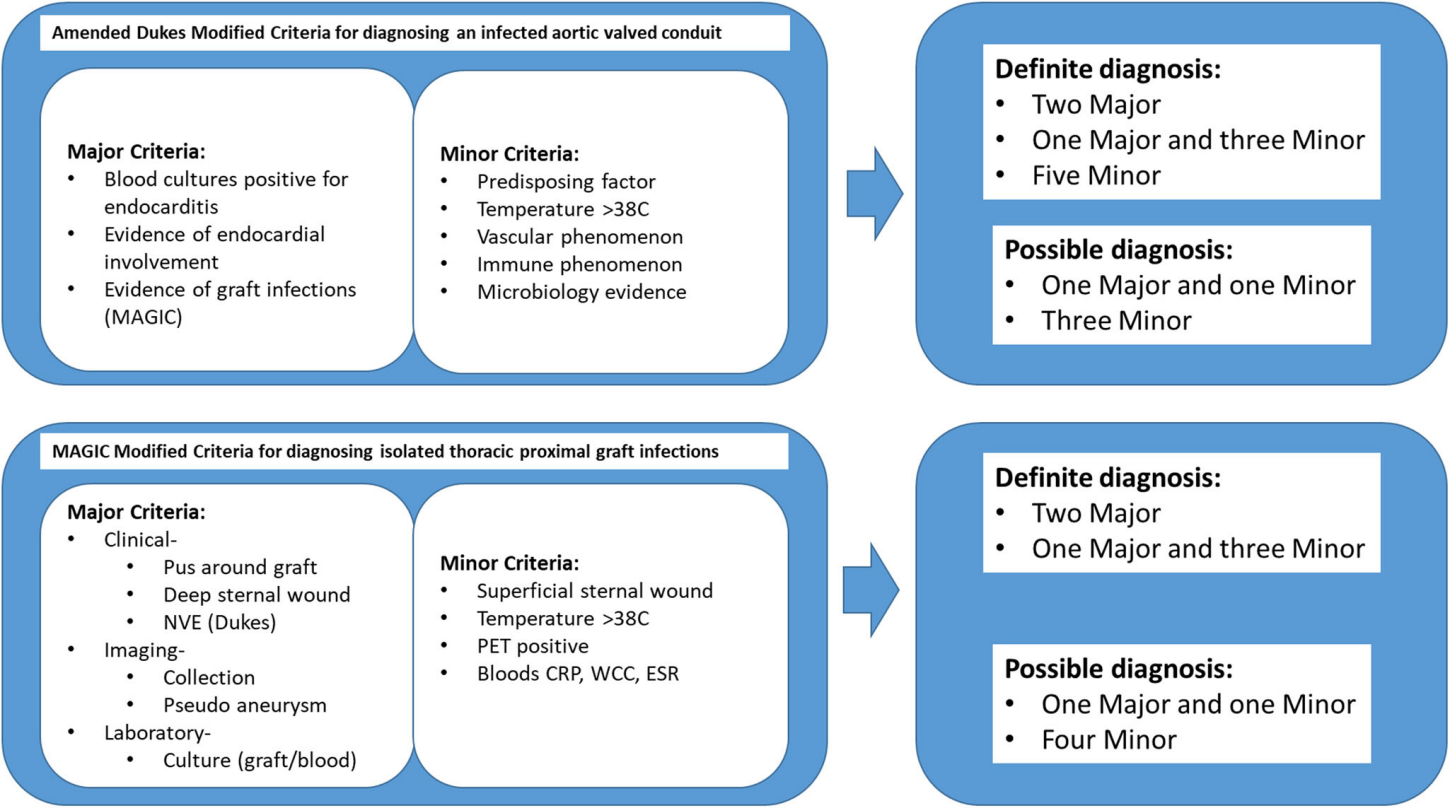
Diagnostic scheme

The management of aortic graft infections collaboration team has set out criteria for diagnosing vascular graft infections.^7^ The approach is for patients presenting under the care of Vascular Surgeons within the United Kingdom and therefore largely exclude patients with thoracic aortic graft infections under the care of cardiac surgeons. We latterly have formally modified their approach to include nuances of thoracic aortic grafts (Figure 1) however an emphasis on purulence, mechanical dehiscence, and positive cultures characterized the diagnostic process throughout the study period. The diagnostic emphasis is on major and minor criteria within three domains of clinical, imaging, and laboratory criteria. The main modification from the original MAGIC criteria includes Major clinical findings of sternal wound with graft exposure and endocarditis of valve prosthesis.

#### 2.1.2 Proximal graft and prosthetic valve infections

Dukes modified criteria^6^ was used to diagnose endocarditis of the prosthetic valve, with the features described within the modified MAGIC criteria describe above, as additional major criteria, in diagnosing infection of a valved conduit (Figure 1).

### 2.2 Management approach

**Figure 2:**
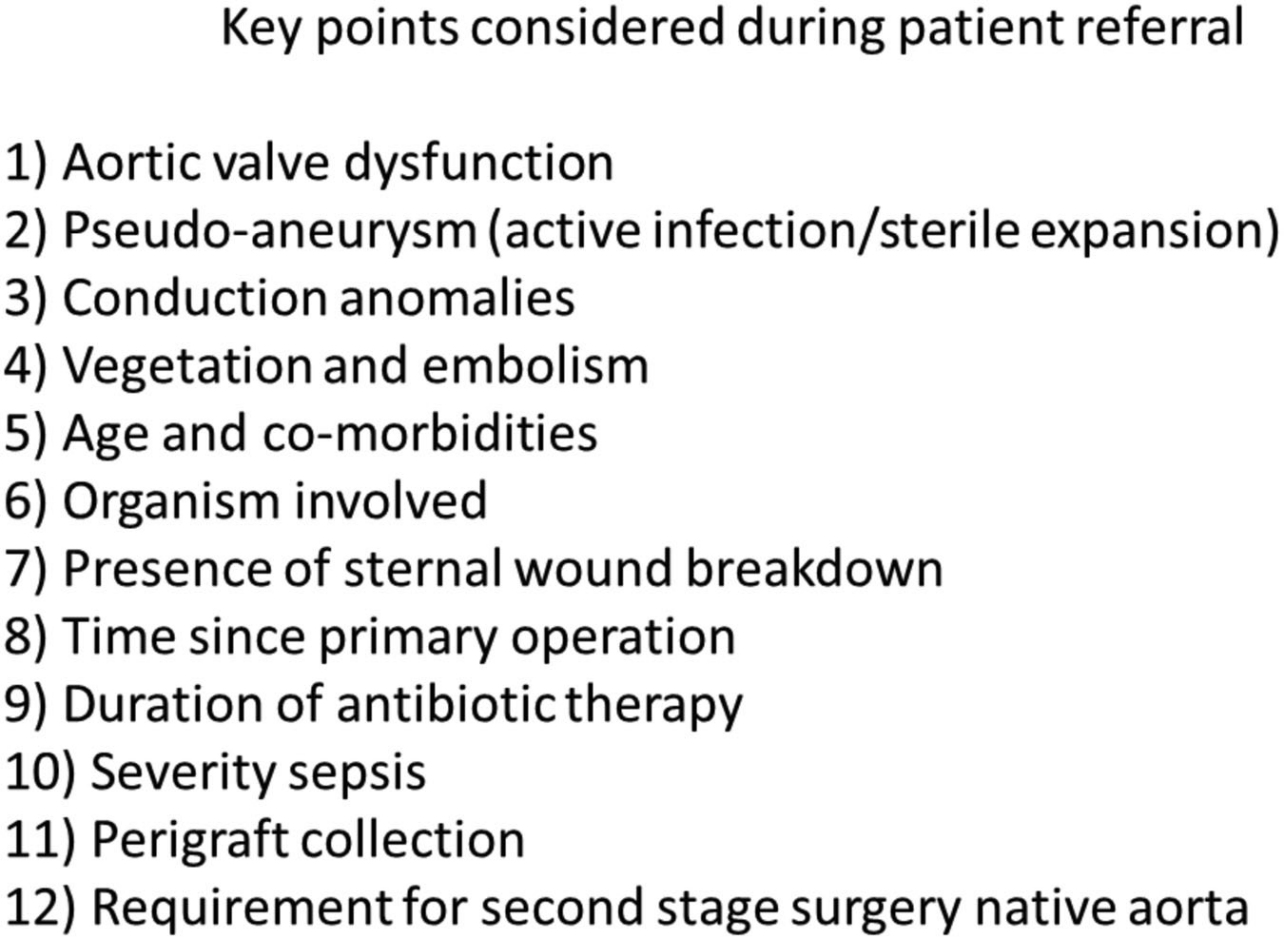
Key features in referrals

**Figure 3:**
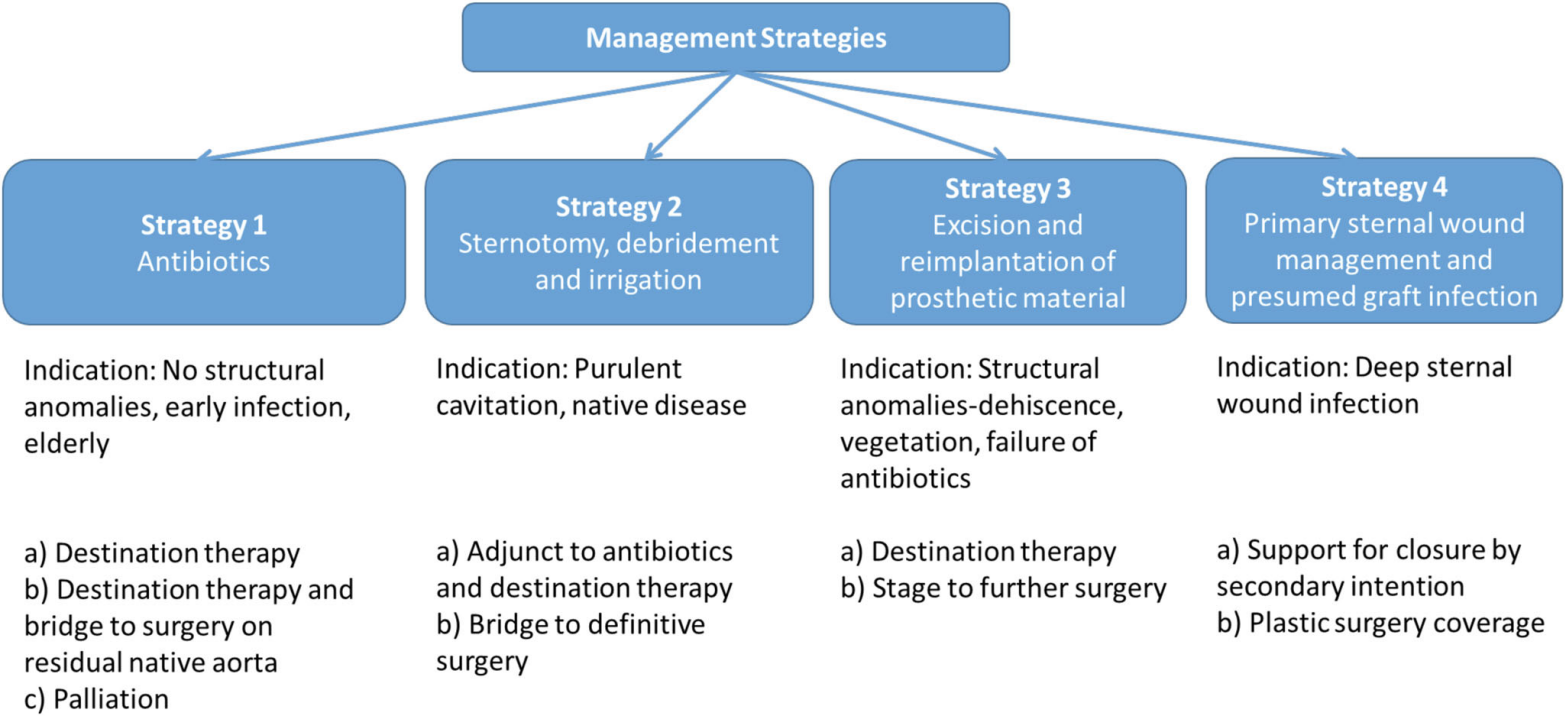
Management strategies

Our approach to the management of patients with infected proximal aortic grafts is influenced by several aspects of the patient (Figure 2) and our strategies are summarized in Figure 3.

#### 2.2.1 Strategy 1: antibiotics

(a) *Destination therapy*. Where ever feasible, dependent on the patient, pathology and organism, our intention was to cure patients of infection with antibiotics alone.
(b) *Destination therapy and bridge to surgery on residual aorta*. Patients with residual native vessel disease beyond infected proximal grafts were treated with antibioitcs before redo surgery, conserving the prosthesis.
(c) *Palliation*. Dependent on patient age, frailty and comorbidities, some patients were managed with antibiotics alone despite pathologies that would have otherwise been indications for surgery such as root abscess, pseudoaneurysms and prosthetic valve degeneration. On occasions this even included permanent pacemeakers for complete heart block.

#### 2.2.2 Strategy 2: sternotomy, debridement, and irrigation

**Figure 4:**
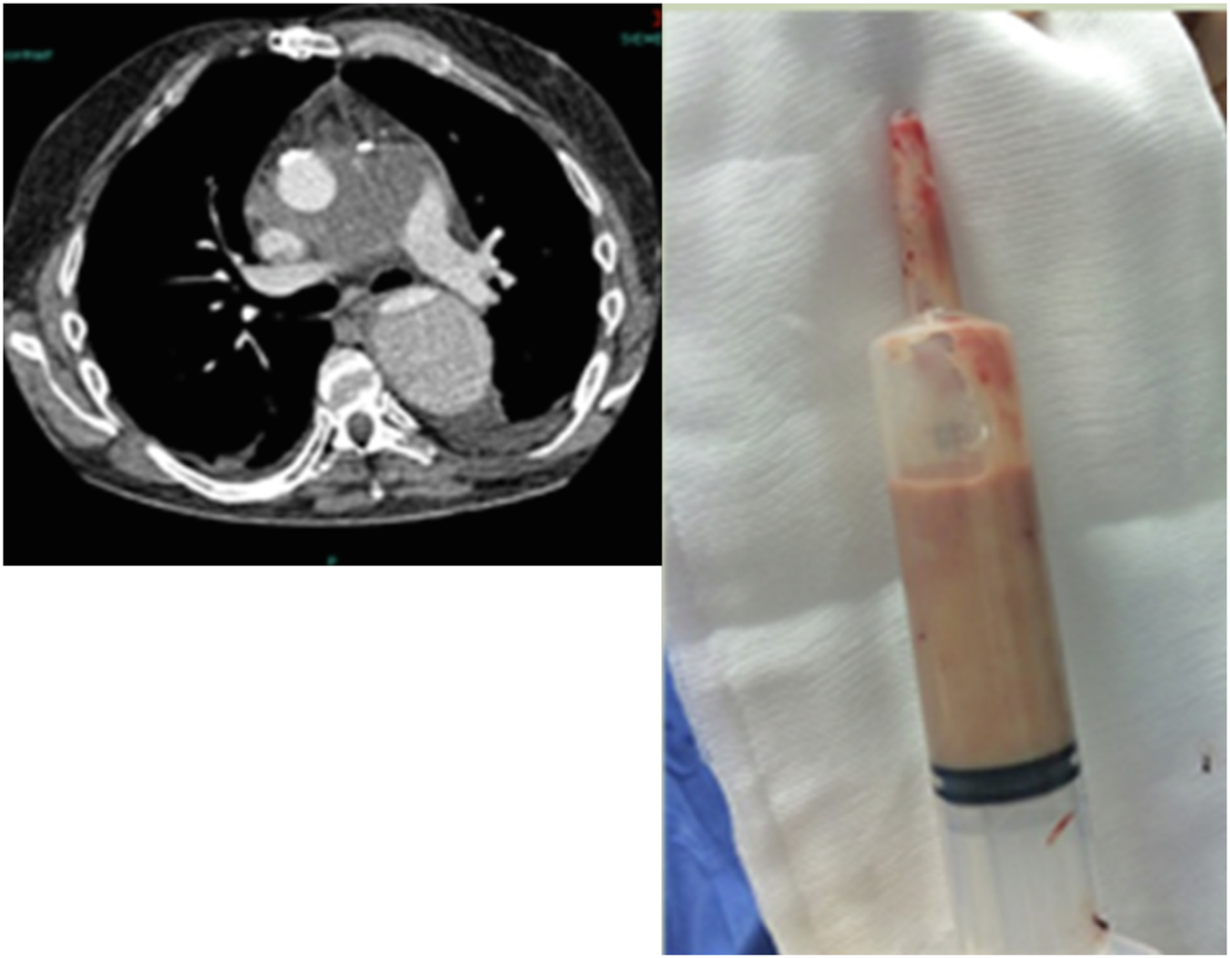
Purulent perigraft collection

(a) *Adjunct to antibiotics and destination therapy*. In the presence of computed tomography (CT) evidence of purulent cavitation around the graft patients underwent redo sternotomy and drainage of pus (Figure 4). A small number of patients had debridement, another procedure and closed but vast majority had an irrigation system set up of Rifampicin (600 mg/L Saline at 100 ml/h) infusing from a system at the top of the sternum and draining through conventional drains exiting through the lower end of the wound. In some patients without any other indication for surgery (pseuodoaneurysm or prosthetic valve degeneration), this approach was used as an adjunct to IV antibiotics with the intention to cure the infection. This irrigation system was continued up to 3 weeks dependent on cultures and patient status.
(b) *Bridge to definitive surgery*. In some patients this approach was used simply to reduce the burden of infectious material before attempting surgical excision of the graft and reconstruction.

#### 2.2.3 Strategy 3: excision and reimplantation of prosthetic material

a) *Destination therapy*. In patients with immediate indications for intervention, such as pseudoameurysms, prosthetic valve failure or failure of medical management, surgery may be the primary and definitive approach.
b) *Stage to further surgery*. Rarely patients with residual native aortic disease were left unoperated in the presence of infected prosthetic grafts, to reduce the burden of prosthetic material.

#### 2.2.4 Strategy 4: Primary sternal wound management and presumed graft infection

a) *Support for closure by secondary intention*. For some patients with sternal wound breakdown and deep mediastinal infection, but without hard indications for redo surgery, the approach may include measures to encourage healing such as VAC pump treatments.
b) *Plastic surgery coverage*. For patients with sternal wound breakdown and graft exposure, treated successfully with antibiotics, but with failure to close the defect, reconstructive techniques, such as pectorial rotation may be used.

### 2.3 Generality of surgical methods

Patients undergoing redo surgery were treated in a variety of ways in terms of redo‐sternotomy, bypass and operative procedures which commonly involved complex root reconstructions with a variety of prosthetic roots including homografts and Cabrol reconstructions.

## 3 RESULTS

We performed a total of 2079 aortic procedures via sternotomy between the period of 1998–2019. Of these, 173 were redo aortic procedures and 20 were for infected proximal aortic grafts. A total of 26 patients presented with proximal graft infections and were treated accordingly.

### 3.1 Demographics

The mean age of the cohort was 57 ± 11 years. For patients with a primary non‐surgical strategy median age was 62.4 years and for those with a primary operative strategy was 56.4 years.

### 3.2 Diagnosis

All patients presented with signs and symptoms of sepsis including general malaise and at least one major and three minor criteria (Figure 1). Bacteria were isolated from blood cultures in 25 of the 26 patients. 69% were identified as staphylococcal aureus. Twelve percent of patient has valvular dysfunction, 54% patients had pseudoameurysms and 19% patients had peri‐graft collections. Of the five patients with purulent collections, no positive cultures were isolated from this fluid. A triad of blood cultures, echocardiography and CT formed the principle modalities in diagnosis in all cases. Positron emission tomography CT was not found particularly useful in anything other than identifying sites of secondary infection.

### 3.3 Strategies

Thirteen out of 26 patients (50%) underwent a primary strategy of explant and surgical repair. These patients were those deemed fit and with hard indications such as root abscess, pseudoameurysms, valvular dysfunction or uncontrolled sepsis. Ultimately, seven patients crossed over into the surgical arm with 20 of the 26 patients undergoing reconstruction. Five patients were identified as having peri‐graft collections and were felt would benefit from drainage as either an adjunct to definitive antibiotic treatment or as a stage to definitive surgery.

### 3.4 Operative approach

Patients required a range of approaches for cannulation dependent of CT appearances including central and peripheral bypass, with and without deep hypothermic circulatory arrest. Explant of infected material was typically challenging requiring Cabrol grafts to the left and right (75%) coronary buttons with redo Bentall procedures. A range of prostheses were using including mechanical valve conduits (9/16, 56%) and biovalve conduits (7/16, 44%). Given destruction of the left ventricular outflow tract (LVOT), use of deep semi‐continuous 2/0 Prolene was required to secure the conduit in all cases of redo Bentall. Table 1 is summary of our study outcomes.

**Table 1:** Summary of our cohort demographics, microbiology, results and outcomes

	**Total cohort (*n* = 26)**
Mean age (*SD*) years	57 ± 11
Male (%)	23 (88%)
Aortopathy (%)	
Marfan	4 (15)
Bicuspid aortic valve	8 (31)
Unknown	14 (54)
Previous surgery (%)	
AVR	3 (12)
Ross	1 (4)
Ascending aorta for type A dissection	1 (4)
Homograft	1 (4)
Valvotomy	1 (4)
None	19 (73)
Latest surgery before admission (%)	
Mechanical root replacement	14 (54)
Bio‐root	9 (34)
Ascending aorta and AVR	1 (4)
Ascending aorta (%)	1 (4)
Total arch and FET	1 (4)
Interval in months (*SD*)	42.5 + /− 35.8
Microbiology (%)	
Staphylococcus aureus	8 (31)
Coagulase negative staphylococcus	4 (15)
Streptococcus equisimilis	1 (4)
Methilicin resistant staphylococcus aureus	2 (8)
Streptococcus mitis	1 (4)
Enterococcus faecalis	2 (8)
Streptococcus epidermidis	1 (4)
Staphylococcus Warneri	1 (4)
More than one organism	3 (12)
Streptococcus agalactiae	1 (4)
Streptococcus viridans	1 (4)
Unknown (possibly culture negative)	1 (4)
Associated valve dysfunction (%)	3 (12)
Pseudoaneurysm (%)	14 (54)
Heart block (%)	1 (4)
Peripheral vegetation/embolism (%)	7 (27)
Sternal wound infection (%)	2 (8)
Purulent cavitation (%)	5 (19)
Resternotomy and irrigation (%)	5 (19)
Medical management (%)	6 (23)
Diagnosis criteria (%)	
1 or more major criteria	26/26 (100%)
3 or more minor criteria	26/26 (100%)
Surgical management (%)	20 (77)
Ascending with total arch	2/20 (10)
Mechanical root replacement	9/20 (45)
Bio‐root	7/20 (35)
AVR, ascending and hemiarch replacement	1/20 (5)
Ascending	1/20 (5)
Bentalls: at least 1 Cabrol	15/16 (94)
Bentalls: bilateral Cabrol technique	12/16 (75)
Resternotomy and irrigation (%)	5/26 (19)
In‐hospital mortality (%)	5/26 (19)
Hospital survival (%)	21/26 (81)
Overall operative survival (%)	15/20 (80)
1 year survival (%)	18/26 (69)

Abbreviations: AVR, aortic valve replacement; FET, frozen elephant trunk.

## 4 DISCUSSION

Patients presenting with proximal aortic graft infections are a challenge for clinicians.^8^ Outcomes are poor and published data are limited and mostly only from recognized major aortic centers, reflecting the experience and skill sets required managing these patients. Coselli et al.^2^ argued that while antibiotics are the cornerstone of treatment of infected grafts, this is rarely successful without surgical intervention, commenting on the study by Akowuah et al.^3^ The authors suggest the question is only whether the graft can be salvaged through debridement and irrigation or whether explant and reconstruction is required. Their attempt of medical therapy alone in seven patients had 43% failure rate.^2^ Of their series of patients with infected Bentall procedures, 5 of their 11 patient died within 30 days (46%). In our experience, the decision for antibiotic therapy or surgical intervention is not a binary decision but dependent of patient factors and extent of disease. Some other authors report marginally better outcomes in some sub‐groups,^4^, ^5^ however re‐doing a Bentall root replacement in the presence of infection is a formidable challenge.^9^, ^10^

Liverpool Heart and Chest Hospital has developed a systematic approach to the diagnosis of late presenting, post primary hospital discharge, proximal aortic graft infections (>3 months) and a step wise strategy in managing patients. This manuscript presents our current series of patients with this approach and adds to the current limited international data. Unlike other recent series,^4^, ^5^ we have excluded patients with index admission, very early suspicion of prosthetic infection which in our experience present complex signs and symptoms related to multi‐factorial postoperative process and are exclusively treated successfully with IV antibiotics alone.

### 4.1 Diagnostic pathway

There are currently no specific diagnostic approaches to proximal thoracic aortic graft infection whether isolated or with associated prosthetic aortic valves. We have used a combination of Dukes (Durack et al.^6^) and MAGIC (Lyons et al.^7^) criteria to produce a weighted scheme for diagnosing infection in isolated proximal thoracic graft infections and valved graft conduits (Figure 1). While this diagnostic approach helps provide some objectivity, in both approaches the presence of a positive blood culture and structural pathology, of valve or graft, are the principle criteria. In our series all but one had positive blood cultures (96%), forming the commonest major criterion, of which 69% were staphylococcal in etiology. Other major criteria were from a mixture of pseuodoaneurysm (14/26, 54%), purulent cavitation (5/26, 19%), and valvular dysfunction in only 3 out of 26 (12%) patients. While early postoperative infection within the index hospital admission is challenging, for patients representing unwell following discharge, the diagnosis was usually apparent. The literature is consistent in reporting Staphylococcus Aureus as the main causative organism in most such infections^5^ and as such associated with extensive tissue destruction.

### 4.2 Choosing a strategy: what works?

There are key features in the presentation (Figure 2) that help guide us in choosing a strategy (Figure 3). As stated above, antibiotics are the main stay. The four principle drivers of strategy are patient age, frailty, structural pathology and sternal wounds. We attempted antibiotics as a single modality approach in six patients. Treatment with antibiotics in isolation was chosen as a strategy with two groups of patients. First, in fit and healthy individuals with no hard indications for surgery and with the intentional outcome of successful treatment with antibiotics stopped. The second group was characterized by patients who were elderly, frail, co‐morbid, with and without structural defects. The intention in this group was discharge home, either for palliation or long term management on antibiotics. Interestingly even some patients with a root abscess or pseuodoaneurysm were successfully managed into a sterile chronic state. Three of these six patients went on to have surgery (3a) and three continued on antibiotics treatment and were alive a year later. This success is in broad agreement with other published series.

A number of surgical based strategies were adopted dependent on presentation, either re‐sternotomy and drainage of collections as a definitive strategy, as a staged bridge to definitive explant of infected material, or re sternotomy and explant as a primary strategy. Purulent peri‐graft collections (Figure 4) are a barrier to successful antibiotic treatment and pose significant issues in definitive explant. With such a high infective burden the risks are of massive systemic inflammatory response syndrome response on cardiopulmonary bypass and potential for reinfection of newly implanted grafts. Interestingly however in our series we failed to culture any organisms from this fluid in any of the patient. Five patients underwent re‐sternotomy, irrigation as adjunct (2a) to definitive antibiotic treatment (2/5), while two out of five underwent further definitive surgery after irrigation. One patient was deemed too frail to undergo further surgery. Two of these patients required explant of previously placed presternal pericardial patches. Attempted salvage of grafts is a common theme in the existing literature particularly in the presence of sternal wound infections. Akowauh et al.^3^ published a series of eight patients with proximal graft infections principally with sternal wound breakdown (6/8 patients) with five undergoing debridement and conservation of the graft, the group advocating this approach. Given the mortality of redo Bentall procedures in our series and what is published in literature,^2^, ^5^, ^11^ it is not unreasonable to attempt antibiotic therapy with or without drainage, debridement and irrigation as a lower risk procedure but accepting the approach may fail in roughly half of patients. Umminger et al.^4^ published an attempt to compare strategies of graft replacement versus preservation in this group of patients. In a series of 25 patients they compared 11 patients treated by graft replacement versus 14 where debridement and irrigation were attempted. The approach was unusually aggressive in that all were early graft infection of less than 1 year. The group concluded that washout and irrigation of infected grafts was best performed within 4 weeks of surgery while for those diagnosed between 3 and 6 months, graft replacement was preferred. The groups were not entirely compatible. In our experience all such very early potential infections (<4 weeks) are treated successfully with antibiotics alone. A multi‐center study involving 68 patients from six centers in Japan focused on the benefits of pedicled grafts (omental and muscle) as an adjunct to treatment but again the series included a high proportion of very early, index hospital infections (43/68, 63%).^5^ Of the 68 patients, only 18 underwent explant with the rest undergoing irrigation with or without flaps or antibiotics alone. These strategies were all associated with a high mortality between 26% and 55%. We have not used flaps in any of our series of patients and have no reported reinfections.

Resternotomy and redo surgery as a primary strategy was the main approach in our series (13/26. 50%), however seven patients with other primary strategies eventually crossed over into a surgical strategy (77%). Explanting and re‐performing a Bentall root replacement, often in the presence of extensive tissue destruction in the LVOT and around the anterior mitral leaflet and fibrous trigonal area is a surgical challenge. Of the 16 Bentall procedures, 14 were in patients who had had previous Bentall operations. All five deaths were within 24 h and in the operated redo Bentall Group giving a mortality of 31%. Although the Group performed six “Commando” or “UFO” procedures for endocarditis, surprisingly none were for prosthetic valve endocarditis of a previous graft.^12^ For these reasons and the associated mortality risk, the strategy is reserved for patients with hard indications. In the context of our series we have used a number of valves, grafts, and valved conduits dependent on patient factors and availability of prostheses. Several technical features of redo surgery were consistent due to the LVOT destruction and the adhesions around the existing coronary buttons. All our cases, irrespective of conduit, required deep semi‐continuous suturing of the conduit to the LVOT. Nearly all cases required Dacron 10 mm Cabrol grafts to the coronary ostia.

In summary the key message in our data is that starting with strategy 1 (antibiotics), for any patient other than palliation, will be unsuccessful as a definitive treatment in 75% of the cohort. Irrigation as an adjunct to antibiotics and definitive treatment (3a) was successful in just two patients out of five, eventually coming off antibiotics all together however as an adjunct to further surgery (3b), the strategy worked in 2. Primary graft explant (3a) was the commonest approach and the commonest default after failed other strategies. Our experience suggests we can attempt to treat sepsis, drain collections and manage wounds but in the end most patients will end up with explant of the infected prosthesis. These strategies are not binary decisions but often evolve over time.

### 4.3 Postoperative management

Patients have routine predischarge CT aorta and echocardiography to act as baseline imaging. Antibiotic administration regime and duration are based on the organism, intraoperative findings and clinical progress. As a routine patients get between 6 and 12 weeks postoperative parenteral therapy with weekly c‐reactive levels (C‐reactive protein [CRP]) and further imaging at the end point with a decision on further treatment. This is in disagreement to recent data suggesting that in patients with endocarditis, patients only require two weeks antibiotics following their last positive blood culture.^13^ Weekly CRPs are maintained for 6 weeks following cessation of antibiotics. Finally, such patients remain under lifelong surveillance.^14^

## 5 CONCLUSION

Adopting a systematic and flexible patient specific approach to the diagnosis and management of patients with proximal aortic graft infections results in reasonable (69%) at 1 year survival. In the majority of patients' surgery is ultimately required to achieve a curative treatment however this comes with high operative mortality risk.

## CONFLICT OF INTERESTS

All authors declare that there are no conflict of interests.

## AUTHOR CONTRIBUTIONS

All authors reviewed and approved the final manuscript.
